# The Importance of Tumor Stem Cells in Glioblastoma Resistance to Therapy

**DOI:** 10.3390/ijms22083863

**Published:** 2021-04-08

**Authors:** Vincenzo Mattei, Francesca Santilli, Stefano Martellucci, Simona Delle Monache, Jessica Fabrizi, Alessandro Colapietro, Adriano Angelucci, Claudio Festuccia

**Affiliations:** 1 Biomedicine and Advanced Technologies Rieti Center, Sabina Universitas, 02100 Rieti, Italy; f.santilli@sabinauniversitas.it (F.S.); s.martellucci@sabinauniversitas.it (S.M.); j.fabrizi@sabinauniversitas.it (J.F.); 2 Department of Experimental Medicine, Sapienza University, 00161 Rome, Italy; 3 Department of Biotechnological and Applied Clinical Sciences, University of L’Aquila, 67100 L’Aquila, Italy; simona.dellemonache@univaq.it (S.D.M.); alecolapietro@gmail.com (A.C.); adriano.angelucci@univaq.it (A.A.); claudio.festuccia@univaq.it (C.F.)

**Keywords:** glioblastoma, glioblastoma stem cells, cancer stem cells, glioblastoma therapy resistance, glioblastoma stem cells and therapy resistance, new drugs in the treatment of glioblastoma stem cells

## Abstract

Glioblastoma (GBM) is known to be the most common and lethal primary malignant brain tumor. Therapies against this neoplasia have a high percentage of failure, associated with the survival of self-renewing glioblastoma stem cells (GSCs), which repopulate treated tumors. In addition, despite new radical surgery protocols and the introduction of new anticancer drugs, protocols for treatment, and technical advances in radiotherapy, no significant improvement in the survival rate for GBMs has been realized. Thus, novel antitarget therapies could be used in conjunction with standard radiochemotherapy approaches. Targeted therapy, indeed, may address specific targets that play an essential role in the proliferation, survival, and invasiveness of GBM cells, including numerous molecules involved in signal transduction pathways. Significant cellular heterogeneity and the hierarchy with GSCs showing a therapy-resistant phenotype could explain tumor recurrence and local invasiveness and, therefore, may be a target for new therapies. Therefore, the forced differentiation of GSCs may be a promising new approach in GBM treatment. This article provides an updated review of the current standard and experimental therapies for GBM, as well as an overview of the molecular characteristics of GSCs, the mechanisms that activate resistance to current treatments, and a new antitumor strategy for treating GSCs for use as therapy.

## 1. Glioblastoma: An Overview

Among primary central nervous system (CNS) tumors, glioblastomas (GBMs) are the most common and aggressive brain tumors in adults. This neoplasia represents high-grade (WHO grade IV) gliomas. The tumor belongs to the astrocytic lineage. The exact origin is unknown: they may derive from neural stem cells (NSCs) or glial precursor cells, less likely from mature astrocytes [1,2]. GBM cells easily infiltrate into surrounding brain tissue, determining a histopathological inflammatory pattern [3,4] characterized by endothelial necrosis. Local invasion represents a common characteristic of all diffuse gliomas. The diffuse (grade II) shows low proliferation rates while anaplastic (grade III) astrocytomas are characterized by angiogenesis. The high-grade (IV) gliomas, named GBMs, present high proliferation, angiogenesis, and a typical necrotic lesion called “pseudopalissading necrosis”, microvascular proliferations sometimes associated with thrombosis.

The majority of GBMs (~90%) develop rapidly in elderly patients without clinical or histological evidence of previous lesions, and, in this case, we speak of primary GBMs. Secondary GBMs that display isocitrate dehydrogenase (IDH) gene mutation progress from low-grade diffuse astrocytomas in younger patients, with a low degree of necrosis, or anaplastic astrocytomas, preferably localizing in the frontal lobe with a low degree of necrosis and a significantly better prognosis [5,6].

In Italy, every year, GBMs affect approximately 1500 people, 54% of whom are male [7,8], while in the United States in 2016, 12,120 cases of GBM were diagnosed [9]. GBMs can occur at any age, but 70% of cases occur between 45 and 70 years of age [1]. Most GBM patients die within two years despite receiving standard-of-care treatments [10], with a median survival of 15 months from diagnosis in patients undergoing safe maximum resection plus adjuvant chemoradiation [1,11,12] and five-year survival rate of 5% [9]. If left untreated, patients die within months of diagnosis. The prognostic factors involved in survival include age, performance status, grade, specific markers (methylated/unmethylated status of O6-methylguanine-DNA methyl-transferase methylation (MGMT) gene promoter, mutations of IDH 1 and IDH 2 or mutations of the TERT promoter, and epidermal growth factor receptor (EGFR) overexpression), and, possibly, the extent of resection [1,13]. The prognosis is better if IDH1/2 gene mutation is present.

Signs and symptoms, such as headache, nausea, vomiting, and/or drowsiness, may develop when the tumor begins to put excess pressure on the brain [14]. Affected people may also experience other features depending on the size and location of the tumor [15].

The causes of GBMs are currently not well known; possible genetic and environmental risk factors are being investigated, although it is currently known that most are sporadic causes [16]. GBM’s aggressiveness seems to derive from a small fraction of a subpopulation of cancer cells, called glioblastoma stem cells (GSCs), which show functional properties such as multipotency and self-renewal, similar to those of NSCs [17]. GSCs reside in specialized vascular niches in close contact with the cerebral endothelium cells, which modulate self-renewal, determine cell fate, and protect these cells from chemo and radiation therapies (RTs) [18]. Furthermore, it has been proposed that small amounts of GSCs remain in brain tissue after surgical resection, causing rapid tumor regrowth [19] and resistance to radiotherapy and/or temozolomide (TMZ) treatment, contributing to tumor recurrence, aggressiveness, and disease progression [20].

## 2. Heterogeneity of Human Glioblastomas and Glioblastoma Stem Cells

It is known that the heterogeneity of the tumor represents one of the main characteristics in tumorigenesis, being able to determine tumor progression and treatment resistance such as resistance to chemotherapy and radiotherapy. Indeed, tumor clones resistant to the treatments are one of the predominant causes of therapy failure and tumor recurrence [21].

Despite the genetic evolution of cancer cell models of human tumors, they show specific hierarchies related to epigenetic programs and developmental pathways in which cancer stem cells (CSCs) can lead tumor growth and give rise to differentiated progeny [22].

According to the CSC theory, only a cell subset keeps both self-renewal and differentiation capacity.

Regarding brain cancers and the understanding of GBM biology, the basic idea is that tumor initiation, maintenance, and regrowth following treatment are enabled by GSCs [23], as demonstrated by the xenotransplantation of specific GBM subgroups characterized by surface marker expression. Singh et al. described a xenograft assay able to identify initiating cells in human brain tumors that start tumors in vivo. The authors demonstrated that only the CD133^+^ brain cancer fraction contains cells with the ability to start cancer in mouse brains. Indeed, the injection of a few CD133^+^ cells produced a tumor that could be transplanted in succession and was a phenotype copy of the original tumor in the patient, while CD133^−^ cell injection did not cause a tumor [24].

Moreover, thanks to a genetically engineered mouse model of glioma, Chen et al. described a subpopulation of endogenous tumor cells. The authors reported that, after the drug TMZ’s administration to transitorily arrest tumor growth, this kind of cell gives rise to a new tumor cell. According to the CSC hypothesis, Chen et al. affirmed that, in their model, only CSCs were able to sustain tumor growth, and they were the cause for recurrence after treatment failures in their model [25].

More recently, single-cell RNA-sequencing analysis has been performed in order to profile primary G cells.

Patel et al. performed single-cell transcriptomics in order to characterize gene expression programs in cells from five primary GBMs. They found that the cells changed their expression of diverse transcriptional programs related to proliferation, oncogenic signaling, hypoxia, and immune responses. The authors also reported that in vivo tumor cells showed several stemness and differentiation states in addition to variable proliferative capacity and the variable expression of quiescence markers—all of which complicate the therapeutic strategies [26].

Using RNA sequencing (RNA-seq), Tirosh et al. characterized individual cells from different mutant oligodendrogliomas in humans. They observed that several cancer cells were differentiated in two specialized glial plans, while a limited subset of cells was undifferentiated and associated with an NSCs expression program. Also, the authors profiled the developmental programs from genome-wide expression signatures, suggesting that the structure of oligodendroglioma was primarily dictated by developmental programs. According to a model in which CSCs were mainly responsible for sustaining the growth of oligodendroglioma, Tirosh et al. concluded that the most primitive and undifferentiated population of cancer cells could be the major source of proliferating cells in oligodendroglioma [27]. The existence of stem cells in human brain tumors was first demonstrated after isolating clonogenic precursors that gave rise to neurospheres, from postsurgical specimens of human medulloblastoma and GBM [28]. In vitro, GSCs have a high regenerative ability and the capacity to differentiate into glial or neuronal cells [29], expressing specific neural markers, such as CD133, A2B5, L1CAM, SOX2, CXCR4, CD15, CD44, Integrin α6, CD36 [30,31,32,33,34], and generate heterogeneous populations that participate in tumor propagation, drug resistance, and relapse [35]. Different independent works reported the in vivo presence of these self-renewing, tumorigenic GSCs [36] may dedifferentiate and convert into cells with malignant characteristics, playing a critical role in tumor propagation [35]. GSCs are located in hypoxic or anoxic vascular niches in close contact with brain endothelial cells [37], which regulate the phenotypic and molecular profile of GSCs affecting the response to the therapy [19,38]. Reciprocal interactions among GSCs require the activation of multiple pathways that promote tumor propagation including metabolic reprogramming for the hypoxia niche and autophagy pathway, Notch signaling, basic fibroblast growth factor (b-FGF), secretion of vascular endothelial growth factor (VEGF), stromal cell-derived factor 1 (CXCL12) with activation of the membrane-associated receptor CXCR4 [39,40], and activation of hypoxia-inducible factor (HIF2α) [41,42].

### 2.1. Markers Shared between Glioblastoma Stem Cells and Glioblastoma Cells

The identification of novel markers and the targeting of GSCs are fundamental issues in the development of innovative strategies for GBM eradication. The identification of specific molecular targets expressed by GSCs could improve our capacity to diagnose and target more aggressive forms of GBM. Integrated cancer genomics transformed the understanding of signaling pathways downstream of surface receptors. This has encouraged current therapy approaches to capitalize on targeting key oncogenic signaling nodes downstream of a limited number of surface markers.

#### 2.1.1. Erythropoietin-Producing Hepatocellular Carcinoma Receptors

As for other cancers [43], Eph (erythropoietin-producing hepatocellular carcinoma) receptor family members are reported as key molecules in GBM development. These receptors are primarily expressed in early development and are crucial for embryonic development and regulating processes such as cell adhesion and migration [44]. The expression of Eph receptors is very low in adult and differentiated tissues. Conversely, Eph receptor expression becomes upregulated in glioma, driving tumorigenicity and stemness, and has been proposed as a therapeutic target and innovative molecular marker since it is strongly overexpressed in GBM cells but not in normal brain [45].

#### 2.1.2. Src Family

GSCs and GBM cells utilize and express different Src family members of protein tyrosine kinases (SFKs), including Src, Fyn, Yes, and Lyn, and their effects do not always seem to be functionally superfluous. Inhibition of Src family activity in GSCs, responsible for GBM lethality and primary GBM cells, reduces tumorigenicity and boosts survival in preclinical models [46,47,48].

#### 2.1.3. Cannabinoid Receptors

Among different cannabinoid receptors (CBRs), the levels of CB1R and CB2R are only upregulated in some cancer cells without automatically being expressed in the origin tissue type. Significant modifications in the cannabinoid balance system between the levels of endogenous ligands and their receptors develop during malignant transformation in various types of cancer [49], including gliomas [50].

#### 2.1.4. Other Markers of Interest

CD133 is a well-studied marker in order to describe intrinsic differences in stemness determinants. Several studies show that the effect of inhibiting the GSCs proliferation was associated with the reduction of the ratio of CD133 and the suppression of the GSCs invasiveness [51,52].

Recently, Balça-Silva et al. suggested that nucleolin, a multifunctional protein associated with the internalization of different ligands and activities such as chromatin remodeling, the stabilization of mRNA, and the modulation of its translation, is expressed in both GSC and GBM cells. The authors suggested that nucleolin could be a common potential marker in NANOG-positive GSC, OCT4-, and in the corresponding non-stem GBM cells, as well as in SOX2-positive GSCs [53].

All the data suggest the presence of a relatively quiescent subpopulation of endogenous glioma cells that are responsible for sustaining long-term tumor growth through the production of transient subsets of highly proliferative cells. Thus, the identification of brain tumor initiating cells provides insights into human brain tumor pathogenesis, giving strong support for the CSC theory as the basis for many solid tumors, and establishes a previously unidentified cellular target for more effective cancer treatments. Thus, the comprehension of the mechanisms underlying tumor heterogeneity is a fundamental step in developing better precision treatments, with significant potential improvements, particularly in the notoriously therapy-refractory cancer cases.

## 3. Current Therapeutic Strategies in Glioblastoma

Surgical resection of the tumor represents the primary therapeutic approach. The amelioration of the prognosis clearly follows the grade of efficiency in the extent of resection and compatibly with the preservation of neurological function [54,55]. Unfortunately, the high probability of recurrence of tumor cells within centimeters of the primary mass renders surgery, but also the subsequent radiotherapy, less effective than expected [56]. Thus, surgical resection should be as extensive as possible in order to have a better chance of survival and improved surgical procedures are welcomed. These include the marking of tumor cells with fluorescent dyes (the most widely used is 5-aminolevulinic acid, 5-ALA) [55] and the utilization of intraoperative imaging or functional monitoring. In order to reduce the risk of residual disease, the implantation of biodegradable carmustine-impregnated wafers into the tumor bed was also proposed after near or complete tumor resection. Although this procedure was approved by the US Food and Drug Administration (FDA) for first-line treatment of GBM, the use of carmustine (BCNU) wafers has been shown to marginally improve median survival compared to radiotherapy alone, and thus it remains controversial also in consideration of the potential adverse events [57].

After surgical resection, the standard treatment is represented by fractionated localized radiotherapy at the maximum cumulative dose of 60 Gy delivered over six weeks, with lower doses that are recommended in patients with a low performance status [58]. TMZ has become the standard treatment in concomitance and adjuvant setting with radiotherapy for patients with newly diagnosed GBM [59,60]. The absence of effective better alternatives renders this therapeutic protocol the gold standard in the majority of GBM patients. The use of TMZ without radiotherapy has been demonstrated to be effective only in elderly patients with methylated MGMT gene promoter [61,62]. In fact, the genotoxic activity of TMZ is apparent by the formation of O6-methylguanine DNA adducts, which are repaired by the enzyme MGMT. Indeed, methylation in the promoter of the MGMT gene represents one of the three predictive markers that have been proposed in high-grade gliomas. The other two are mutated IDH and loss of heterozygosity of chromosomes 1 p and 19q. However, the utility of the proposed predictive markers is reduced by the fact that tumors with chromosomes 1 p and 19 q almost always have IDH mutations and MGMT promoter methylation [63]. In addition, although the resistance to TMZ in patients harboring unmethylated MGMT promoter is a consolidated notion, the clinical utility of this genetic analysis remains poor because of a lack of alternative therapeutic options [64].

Although the combination of TMZ and radiotherapy has significantly improved outcomes for GBM, few patients survive beyond five years. In addition, attempts aimed to augment the effects of TMZ and radiotherapy have failed, suggesting the necessity of a radical innovation in the therapeutic approach [65].

In 2011, the FDA approved a new therapeutic modality in recurrent GBM patients that represents an absolutely innovative approach—the tumor-treating fields (TTFields). The significant results demonstrated in clinical trials have prompted the inclusion of TTFields in the updated version of clinical guidelines [66]. TTFields delivers low-intensity, intermediate-frequency (200 kHz), alternating electric fields through transducer electrodes applied to the scalp of the patient [67]. Preclinical studies have suggested that TTFields determines the block of cells in the mitotic phase of the cell cycle interfering with chromosomal segregation. In recurrent tumors, TTFields was demonstrated to be noninferior to chemotherapy but with a better toxicity profile [68]. A subsequent phase III study evaluated TTFields in combination with TMZ during the standard protocol in patients with newly diagnosed GBM and permitted the extension of the approval also for this condition due to the significant improvement in progression-free survival (PFS) and overall survival (OS) [69].

For recurrent tumors after prior chemotherapy, there is no established therapeutic regimen available, and oncologists may apply investigational protocols. However, a percentage of patients are candidates for second surgery, in accordance with the morphological characteristic of the recurrent tumor and the time interval between surgeries. Alternative options include nitrosourea-based regimens, alternative dosing regimens of TMZ, and bevacizumab [70]. Nitrosoureas are DNA-alkylating agents that have revealed to be candidate drugs for brain tumors due to their ability to cross the blood–brain barrier (BBB), and they represented first-line treatment of GBM prior to the demonstration of the superior activity of TMZ. Different nitrosourea drugs are available as therapeutic options for GBM, including BCNU, lomustine (CCNU), nimustine (ACNU), and fotemustine (FOT). Some of these have been evaluated also as monotherapy in clinical trials, demonstrating adequate safety and tolerability profile but with limited PFS and modest improvement in OS [71,72]. A combination of CCNU with procarbazine and vincristine may represent another option in recurrent tumors, even if its activity was not significantly different from TMZ treatment [73].

In 2016, bevacizumab was approved by the FDA (to date, not in the European Union) for use as second-line GBM therapy. Bevacizumab is a humanized monoclonal antibody directed against circulating VEGF. Bevacizumab can be considered a salvation option after radiotherapy and chemotherapy failure. Clinical trials have demonstrated that bevacizumab determined a significant improvement in patients with recurrent disease, ameliorating response rates and six-month progression-free rates [74,75]. An improved PFS was also noted when bevacizumab was evaluated as part of standard first-line therapy; however, the data from OS did not justify an inclusion in the routine therapeutic protocol [76]. A significant difference in PFS, but not in OS, was also observed in a European Organization for Research and Treatment of Cancer (EORTC) clinical trial when bevacizumab was added to CCNU [77].

## 4. Novel Anticancer Strategies Targeting Glioblastoma Stem Cells

An important aspect of GBM is the ground and abnormal tumor vessel blood supply in which multiple subclonal driver mutations create a highly adaptable entity that is resistant to all therapeutic approaches. Several studies suggest that GSGs can be responsible for the high recurrence rates in GBM and for the failure of treatment [78]. Moreover, since GSCs represent a considerable target for treating GBM, their elimination is crucial. The main strategy for treating GSCs is based on their direct ablation by targeting cell surface markers. Therefore, specific pathways required for maintaining GSC stemness have also been investigated. However, it has been increasingly acknowledged that another way to specifically target GSCs is to alter the ability of GSCs to interact with their microenvironment through the modulation of the immune system, stroma, and vasculature. We address specific novel signaling pathways that may be targeted to obtain this aim.

### 4.1. Targeting Notch and Sonic Hedgehog Pathways

Notch signaling is an evolutionarily well-conserved pathway involved in many aspects of differentiation, proliferation, and apoptotic events, even though its specific role is highly context-dependent [79]. Notch signal inhibitors might be good candidates for the treatment targeting GSCs [80]. Besides, Notch and some of its downregulation ligands (i.e., Delta-like-1 and Jagged-1) lead to a decrease in the oncogenic potential of GSCs, demonstrating its key role in GSC survival and proliferation (Table 1) [81]. For example, Fan et al. experienced decreased proliferation, increased neuronal differentiation, lowered CD133^+^ cell fraction in vitro, and diminished tumorigenicity in vivo while Notch activation was prevented by γ-secretase inhibitors (GSIs) [82]. On the other hand, pharmacological Notch inhibition may stimulate protective autophagy in glioma neurospheres, resulting in chemoresistance, but combination treatment with the autophagy inhibitor chloroquine could cancel this effect [83]. Functional interaction between Notch signaling and leptin in GBM has also been reported, and leptin seems to enhance the invasive potential of GSCs [84]. Subsequently, they reported that increased expression of leptin receptor (Ob-R) seems to cause TMZ resistance by the increase of stem/progenitor cell features [85]. Also, Panza et al. showed that leptin seems able to directly sustain GSCs features in GBM. They observed an increase of leptin receptors expression in tumor spheres from GBM. Moreover, leptin led to increased efficiency of neurosphere formation and capacity of self-renewal. Thus, leptin can lead to the upregulation of the Notch 1 receptor and to the activation of its downstream effectors and target molecules, suggesting leptin/Notch crosstalk as an alternative therapeutic target for GBM treatment [86]. Moreover, Notch signaling is involved in maintaining GSCs within the hypoxic niche [87,88]. Several studies suggest that GSCs reside in particular zones known as perivascular niches or perihypoxic niches [89]; here, GSCs are protected against radiotherapy and chemotherapy. Others hypothesize the existence of peri-immune niches and/or niches of GSCs in the extracellular matrix (ECM) [90], but all of them can be considered as a portion of the overall GSC niche model in which the activation of Notch has been delineated. For example, Man et al. suggested that the molecule vasorin can act to trigger Notch signaling under hypoxic conditions and identify another potential therapeutic target against glioma [87]. Together with Notch, a high expression of the Sonic hedgehog (Shh) pathway has also been noticed in GBM cell lines and in GBM-derived neurospheres suggesting the Shh inhibitor as a new therapeutic strategy for GBM patients. The authors demonstrated that Shh pathway inhibition by cyclopamine determined a decrease in GBM-derived neurospheres with depletion of stem-like cancer cells in GBM. Moreover, the GBM cells injected intracranially were no longer able to form tumors once treated with an inhibitor of Shh. Cyclopamine has been found to affect GBM cell lines but also sensitize them to TMZ treatment. In a first-line study, treatment with Shh inhibitor followed by TMZ on GBM-exposed cell lines resulted in a reduction in GBM viability. Moreover, cyclopamine potentiated TMZ treatment, inducing apoptosis through the activation of caspase-3 cleaved [91].

### 4.2. Targeting Wnt Pathway

The Wnt signaling pathway includes several Wnt ligands, Frizzled (FZD) receptors, and numerous coreceptors. The Wnt binding to the FZD–LRP5/6 receptor complex on the cell surface of receiving cells leads to activation of the Wnt/β-catenin pathway, also called canonical Wnt pathway [98]. The activation of this pathway results in β-catenin proteins accumulation and nuclear translocation where they regulate context-dependent expression of Wnt target genes. Wnt and their downstream effectors act as key molecules in regulating proliferation, migration, and cell fate decision. Thus, it is easy to understand that Wnt signaling deregulation can be involved in the onset of various cancers including GBM. For example, a study demonstrated a contribution of pleomorphic adenoma gene-like 2 (PLAGL2) into GBM stemness triggering of the canonical pathway, such as Wnt6, FZD9, and FZD2, confirming a PLAGL2 overexpression in GBM [92]. Also, Rheinbay found widespread activation of genes normally repressed in GSCs that was dependent on the chromatin state [93]. Besides, Adamo et al. demonstrated a significant overexpression of receptor-like tyrosine kinase (RYK) in GSCs, reporting a role of this kinase in influencing stemness frequency, cell migration, and invasion in GBM through β-catenin stabilization [94]. On the other hand, activated Wnt/β-catenin signaling has been observed only in a small proportion of GBM cells that showed a gene expression profile correspondent to the GBM subgroup in which there was also an elevated expression of Achaete-scute homolog 1 (ASCL1). Double inhibition of the Notch and Wnt pathways in this GSCs subgroup seemed to decrease timing and increase the extent of neuronal differentiation, indicating new therapeutic opportunities for development of combined treatments. The targeting efficacy of Wnt/β-catenin pathways has been also noticed by Rheinbay et al. [93]. These authors emphasized the importance of GBM associated-endothelial cells (ECs) in tumor chemoresistance acquisition and suggested the existence of a resistance mechanism based on ECs transformation and stemness activation. They also reported that mesenchymal transformation was triggered by Wnt/β-catenin signaling activation, confirming the importance of this targeting to overcome pharmaco-resistance in GBM. Lastly, a new study pointed to the role of epigenetic modifications in the regulation of the Wnt pathway assuming that the different patterns of microRNAs (miRNA/miR), such as miR-138-2-3p and miR-770-5p, may carry out a key role in the development and progression of GBM [99].

### 4.3. Therapeutic Targeting of the Tumor Microenvironment

The tumor microenvironment (TME) comprises a large set of cancer and noncancerous cells inside the tumor such as GSCs, fibroblasts, immune cells, microglia/macrophages, ECs, and vascular pericytes. It also includes the ECM, growth promoting and inhibiting factors, hormones, nutrients, chemokines, produced by all cell types within the TME. [100]. The main role of the TME is to produce and control a dense network of autocrine and paracrine signals. This complex network of signaling involving exosomes, tumor–stroma interactions, and ECM factors that vary over time and space in relation to tumor progression modulates gene expression to control signaling pathways. Consequently, changes in gene expression and nongenetic factors both induce individual cells, such as CSCs, to acquire plasticity by showing a variety of phenotypic states, which are pivotal to sustain tumor growth and metastasis, and also to induce therapeutic resistance [101]. These phenotypic states can be transiently acquired by some individual cells within the population, implicating the dynamic regulation of phenotypic heterogeneity depending on local signals influencing a space-specific generation of these cell states. For example, TGFβ, IL-6, exosomes, and many other CAF-secreted cytokines and growth factors can drive mesenchymal transitions [101]. Moreover, the signaling milieu of the hypoxic niche can induce drug-tolerant epithelial-to-mesenchymal transition states [102]. Finally, CSCs that often enrich perivascular niches seem to favor a slow-growing phenotype and chemo-resistance [103]. Therefore, accumulating evidence highlighted the importance of TME for maintaining GSC stemness, indicating the targeting of the microenvironment as a promising approach for GBM treatment [104]. The microenvironment of GBM consists in part of microvasculature and tumor-associated macrophages (TAMs). The use of bevacizumab or agents capable of regulating angiogenesis in tumors has also been investigated in GBM. In particular, Calabrese et al., treating mice carrying xenografts of U87 glioma cells with bevacizumab, reported a decrease in the number of CD133^+^/Nestin^+^ tumor initiation cells, a fall in microvasculature density, and a decrease in tumor growth [95]. Numerous studies have indicated the importance of TAMs as components of the microenvironment, demonstrating the enrichment of TAMs in GBM. For example, Pyonteck et al. reported that macrophages 2 TAMs may promote GBM tumor growth inducing neovascularization and support tumor facilitating GBM progression [96]. Zhou et al. noticed that GSCs secrete periostin (POSTN), which enables GBM progression by TAM recruitment [97]. On the other hand, silencing POSTN in GSCs resulted in relevant reduction of TAM density, inhibition of tumor growth, and improved survival of mice bearing GSC-derived xenografts. As shown in these studies, the relationship between GSCs and their microenvironment may be considered as an alternate approach to treat GBM.

### 4.4. New Drugs in the Treatment of Glioblastoma Stem Cells

The standard treatment for GBM [105] is largely ineffective, and the average survival of a person diagnosed with GBM is only 15 months. Current therapies against GBM show a high percentage of failure associated with the survival of GSCs that repopulate treated tumors [47]. Some evidence suggests that new therapeutic approaches are required to contrast tumor heterogeneity, drug resistance, and a complex tumor-supporting microenvironment, and improve survival and quality of life. Furthermore, it has been observed that GSCs resist conventional treatment (radiotherapy, TMZ) and do not lead to complete tumor remission. The prognosis for GBM is unfavorable, due at least in part to the lack of an effective drug delivery system across the BBB that prevents efficient passage of cancer therapeutics including small molecules and antibodies [106]. Moreover, we need to develop a new drug delivery system to overcome the BBB. Therefore, nanotechnology (liposome, polymeric nanoparticles, gold nanoparticles, and nano-carbon particles) has become the most promising approach to GSC-targeting therapy. The nanoparticles can be improved to facilitate delivery efficiency of drugs, to enhance the accumulation within the tumors, and to promote the capacity for targeting GSCs [107]. In addition to the study of new drug delivery systems, there is an urgent need to identify alternative therapies that are more effective for GBM; for this reason, several studies on new molecules and drugs are currently underway.

Dumitru et al. showed that cannabinoids are able to inhibit the invasiveness and the stem cell-like properties of GBM tumors, and a phase II clinical trial showed positive results regarding the survival of GBM patients upon cannabinoid treatment (Table 2) [108].

The use of nabiximols for the treatment of multiple sclerosis and of purified botanical cannabidiol has brought the therapeutic use of cannabinoids and endocannabinoids into neurological diseases including GBM [109].

Recently, a new approach based on the use of natural products or plant derivatives as chemoprevention drugs and chemotherapy has been performed. Among the natural molecules, the potential therapeutic effects of curcumin against GBM have been investigated. Curcumin can target multiple signaling pathways involved in the development of aggressive and drug-resistant features of GBM, including pathways associated with GSCs activity [110]. A recent paper studied the effect of resveratrol to evaluate antitumorigenic effects on cell proliferation, sphere-forming ability, and invasion in different GBMs and GSCs. Resveratrol is able to reduce proliferation in U87 gliomas and multiple patient-derived GSC lines. Moreover, since resveratrol inhibited the sphere-forming ability, the authors suggest anti-stem cell effects. Additionally, resveratrol blocked U87 gliomas and GSC invasion [111].

Colapietro et al. elucidated for the first time the antitumor properties of crocetin (saffron carotenoid) in in vivo and in vitro glioma models. The treatment with crocetin of all the tumor cell lines observed (i.e., U251, U87, U138, and U373) exhibited antiproliferative and prodifferentiative effects. Moreover, it was possible to observe deep changes in cell morphology and change in mesenchymal and neuronal markers. In addition, crocetin not only had more major effects than radiotherapy alone, but also similar effects to TMZ [112,113]. Another new natural molecule is the PBI-05204 (a supercritical CO_2_ extract of Nerium oleander). The authors used this new compound to evaluate the antitumor efficacy in in vitro and in vivo GBM cancer models and the efficacy against GSCs. The results suggest the drug induction of tumor cell apoptosis and inhibition of PI3 k/mTOR pathways as well as reduced cancer stemness [114]. An innovative therapeutic approach already found in other tumors, such as melanoma and hematological tumors, seems to be the “immunotherapy of tumors”. Preclinical studies on the use of monoclonal antibodies have shown that treatment with cetuximab alone and in combination with radiotherapy increases in vivo survival [115] and can also completely eliminate tumors in EGFR-amplified Patient-Derived Xenografts (PDX)models [116]. Promising prospects could be personalized therapeutic approaches with the combination of different targeted therapies that can cross the BBB, while maintaining therapeutic concentrations, for the treatment of GBM with benefits for GBM patients [117]. Moreover, various treatments are currently in phases II/III of clinical trials (Table 3).

### 4.5. Potential New Immunological Drugs against Glioblastoma Stem Cells

Another promising strategy is the utilization of immunological drugs, with the finality of selectively eradicating GSCs without affecting normal tissue. Adoptive cell therapy also represents an innovative therapeutic tool for use against solid cancers and has been shown to elicit an effective antiglioma response [118]. In fact, GSCs have been demonstrated to be highly susceptible to T cell-mediated immunity [119], and an abundant infiltration of brain tumors with T cells has been described in early clinical trials of immunotherapy [120]. The tropism of T cells for brain tumors could be enhanced by modifying their surface receptors in vitro. Early results for this method demonstrate that it can be successfully used to target GSCs in GBM patients [121].

## 5. Conclusions

In this review, we have summarized recent studies that have shown the presence in GBM of GSCs showing therapy-resistant phenotype that could explain tumor recurrence and local invasiveness and, therefore, may be targets for new therapies.

The use of new drugs or alternative drug delivery systems could represent an innovative and important strategy in ongoing therapies targeting these GSCs. In addition, we have provided an overview of the molecular characteristics of GSCs, the mechanisms that activate resistance to current treatments, and a new antitumor strategy for treating the GSCs for use as therapies.

## Figures and Tables

**Table 1:** Potential therapeutic targets against cancer stem cells.

Cancer Stem Cell Pathway	Potential Therapeutic Target	Agents	Effects	Target Model	References
Notch pathway	Notch signaling	Notch-1 siRNA, Delta-like-1 siRNA, Jagged-1 siRNA, Delta-like-1 Fusion protein	Downregulation of Notch and its ligands leads to a reduction of oncogenic potential of GSCs	GBM cell lines	[80,81]
	Notch signaling	γ-secretase inhibitor GSI-18	Inhibition of Notch by GSIs increases neuronal differentiation and decreases tumorigenicity	DAOY, PFSK, D283Med, and D425Med cell lines. HSR-GBM1 and JHH520 GBM neurosphere lines	[82,83]
		MRK003			
	Notch + autophagy targeting	MRK003 + chloroquine	Protective autophagy abrogated by combination with chloroquine	HSR-GBM1 and JHH520 GBM neurosphere lines	[83]
	Notch + leptin	GSIs + LFDI peptide (Leu-Asp-Phe-Ile)	High expression of leptin receptors in tumorspheres from GBM	human fetal glial cells SVG p12, human	[86]
				GBM cell lines	
				U-87 MG, T98G	
	Hypoxic tumor cells	vasorin	Vasorin acts triggering Notch under hypoxic conditions	GSCs and non-GSCs from GBM	[87]
Shh pathway	Shh	cyclopamine	Inhibition of hedgehog pathway by cyclopamine inhibited formation of GBM-derived neurospheres	40–60% reduction in the growth of adherent glioma lines	[91]
Wnt pathway	GBM differentiation pathway	Dickkopf-1 (DKK1) Wnt inhibitor suppress PLAGL2	PLAGL2 acts as an oncogene in human GBM regulating Wnt signaling	primary GBM and established glioma cell lines	[92]
	ASCL1	-	ASCL1 and Wnt signaling are connected and collaborate with developmental transcription factors (TFs). They support GSCs’ growth	GSC lines derived from different human tumors	[93]
	RYK pathway	-	RYK promotes stem cell-like and tumorigenic features to glioma cells and are essential to support GSCs	GBM cell line U87MG, AM38, and U251MG cells	[94]
	Wnt/β catenin	-	Tumor chemoresistance acquisition depends on mesenchymal transformation that is triggered by Wnt/β catenin signaling	GSCs	[93]
Tumor microenvironment	Microvasculature and TAMs	Erlotinib and Bevacizumab	Bevacizumab treatment reduces the number of CD133^+^/Nestin^+^ tumor initiation cells and decreases microvasculature density and tumor growth	CSCs obtained from tumors	[95]
	M2-TAMs and microvasculature	BLZ945-Inhibitor of the CSF-1 receptor (CSF-1R)	TAMs support GBM tumor growth by promoting neovascularization. They play a tumor-supportive role in GBM progression	Proneural GBM	[96]
	TAMs	shPOSTN	Silencing POSTN that recruits TAMS reduces TAM density, inhibits tumor growth, and increases survival of mice bearing GSC-derived xenografts	Human GBM specimens and glioma-derived cells	[97]

**Table 2:** Potential new drugs and treatment against glioblastoma stem cells.

New Drugs and Treatment against Glioblastoma Stem Cells				
Natural Drugs	**Compound**	**Source**	**Effects**	**References**
	Cannabinoids	Cannabis sativa	Inhibits invasiveness and stem cell-like properties of GBM tumor	[108,109]
	Curcumin	Curcuma longa	Targets multiple signaling pathways involved in developing aggressive and drug-resistant features of GBM	[110]
	Resveratrol	Grape skin, blueberries, raspberries, mulberries, and peanuts	Inhibited GBM and GSC growth and infiltration, acting partially via AKT deactivation and p53 induction, and suppressed GBM growth in vivo	[111]
	Crocetin	Saffron	Cotreatment with RTs, similar effect to TMZ	[112,113]
	PBI-05204	Nerium oleander	Induction of tumor cells apoptosis and antitumor effects	[114]
Monoclonal Antibodies	Cetuximab	Erbitux	Cotreatment with RTs for locally advanced disease, or in combination with platinum-based chemotherapy in relapsed and/or metastatic disease	[115,116]

**Table 3:** Some clinical trials in the II/III phase designed for GBM treatment.

Clinical Trials	Treatment	Phase	References
An evaluation of the tolerability and feasibility of combining 5-ALA with BCNU wafers (Gliadel^®^) in the surgical management of primary GBM	5-ALA	II	ClinicalTrials.gov Identifier: NCT01310868 [59,61]
	Gliadel^®^ wafers		
	Radiotherapy		
	Concomitant chemotherapy		
	Adjuvant chemotherapy		
Phase II study of bevacizumab and ACNU in patients with recurrent high-grade glioma	Bevacizumab	II	ClinicalTrials.gov Identifier: NCT02698280 [73]
	ACNU		
Randomized noncomparative phase II trial with bevacizumab and FOT in the treatment of recurrent GBM	Bevacizumab	II	ClinicalTrials.gov Identifier: NCT01474239 [74]
	FOT		
A randomized phase II/III open-label study of ipilimumab and nivolumab versus TMZ in patients with newly diagnosed MGMT unmethylated GBM	Ipilimumab	II/III	ClinicalTrials.gov Identifier: NCT04396860
	Nivolumab		
	NovoTTF-100 A device		
	Questionnaire administration		
	Radiation therapy		
	TMZ		
A prospective, multicenter trial of NovoTTF-100 A together with TMZ compared to TMZ alone in patients with newly diagnosed GBM	NovoTTF-100A device	III	ClinicalTrials.gov Identifier: NCT00916409 [68,69,70,71]
	TMZ		
Phase III trial exploring the combination of bvacizumab and CCNU in patients with first recurrence of a GBM	Bevacizumab	III	ClinicalTrials.gov Identifier: NCT01290939
	CCNU		
	DNA methylation analysis		
	Laboratory biomarker analysis		
Phase III double-blind placebo-controlled trial of conventional concurrent chemoradiation and adjuvant TMZ plus bevacizumab versus conventional concurrent chemoradiation and adjuvant TMZ in patients with newly diagnosed GBM	Radiation therapy	III	ClinicalTrials.gov Identifier: NCT00884741
	Bevacizumab		
	Laboratory biomarker analysis		
	Placebo		
	TMZ		
A randomized phase III open-label study of nivolumab versus bevacizumab and multiple phase I safety cohorts of nivolumab or nivolumab in combination with ipilimumab across different lines of GBM	Nivolumab	III	ClinicalTrials.gov Identifier: NCT02017717
	Bevacizumab		
	Ipilimumab		
Phase III study of standard radiotherapy plus concomitant and adjuvant OSAG 101 (Theraloc^®^) plus TMZ versus standard radiotherapy plus concomitant and adjuvant TMZ patient with newly diagnosed, histologically confirmed GBM multiforme grade IV	Nimotuzumab	III	ClinicalTrials.gov Identifier: NCT00753246

## Abbreviations

GBM	Glioblastoma
GSCs	Glioblastoma Stem Cells
CNS	Central Nervous System
NSCs	Neural Stem Cells
WHO	World Health Organization
MGMT	Methylation O6-methylguanine-DNA Methyl-transferase
IDH1	IDH2 Isocitrate Dehydrogenase Isozymes 1 and 2
EGFR	Epidermal Growth Factor Receptor
RTs	Radiation therapies
TMZ	Temozolomide
CSCs	Cancer Stem Cells
VEGF	Vascular Endothelial Growth Factor
Eph	Erythropoietin-Producing Hepatocellular Carcinoma Receptors
SFKs	Src Family Members of Protein Tyrosine Kinases
CBRs	Cannabinoid Receptors
5-ALA	5-aminolevulinic Acid
FDA	US Food and Drug Administration
BCNU	Carmustine
TTFields	Tumor-Treating Fields
PFS	Progression-Free Survival
OS	Overall Survival
BBB	Blood–Brain Barrier
CCNU	Lomustine
ACNU	Nimustine
FOT	Fotemustine
GSIs	γ-secretase inhibitors
ECM	Extracellular Matrix
PLAGL2	Pleomorphic adenoma gene-like 2
RYK	Receptor-like tyrosine kinase
ASCL1	Achaete-scute homolog 1
ECs	Endothelial cells
TME	Tumor Microenvironment
TAMs	Tumor-Associated Macrophages
POSTN	Periostin
